# Novel Ingredients Based on Grapefruit Freeze-Dried Formulations: Nutritional and Bioactive Value

**DOI:** 10.3390/foods8100506

**Published:** 2019-10-17

**Authors:** Marta Igual, Laura Cebadera, Rosa Mᵃ Cámara, Claudia Agudelo, Nuria Martínez-Navarrete, Montaña Cámara

**Affiliations:** 1 Food Investigation and Innovation Group, Food Technology Department, Universitat Politècnica de València, Camino de Vera s/n, 46022 Valencia, Spain; marigra@upvnet.upv.es (M.I.); clagste@doctor.upv.es (C.A.); nmartin@tal.upv.es (N.M.-N.); 2 Facultad de Farmacia, Departamento de Nutrición y Ciencia de los Alimentos, Universidad Complutense de Madrid, Pza. Ramón y Cajal s/n., 28040 Madrid, Spain; lcebadera@yahoo.es (L.C.); rm.camara@ucm.es (R.M.C.)

**Keywords:** grapefruit, freeze-drying kinetics, microwaves, bioactive compounds, functional food ingredients

## Abstract

Grapefruit is a fruit with interesting nutritional value and functional properties, but a short life. Freeze-drying (FD) is a valuable technique as it produces high-quality dehydrated products. This study is aimed to obtain new food ingredients based on freeze-dried grapefruit formulated with high molecular weight solutes (gum arabic and bamboo fiber) in three different proportions (F1, F2, and F3). To improve the FD, a mild microwave drying pre-treatment was applied. Influence of the water content and the presence of high molecular weight solutes on freeze-drying kinetics was tested by Midilli-Kucuk and Page models. The best FD kinetic model fit on grapefruit powders were Midilli-Kucuk for F2 and F3, and Page for F1, and the adequate freeze-drying times for F1, F2, and F3 were 24, 16, and 18 h, respectively. Final samples were evaluated for nutritional and antioxidant capacity. Gum arabic and bamboo fiber present a protector effect, which results in a significant antioxidant capacity due to the protection of flavonoids and antioxidant vitamins. These novel food ingredients could be of great interest for the food industry in order to develop foods with improved antioxidant capacity as well as enriched in natural fibers and/or micronutrients.

## 1. Introduction

Fruits, independent of their nutritional value, are generally recognized as important sources for a wide array of phytochemicals, which individually, or in combination, may benefit human health, thus are considered bioactive compounds [1].

Citrus fruits are an important source of physiologically active substances that fulfill, like essential nutrients, a beneficial function for health and contribute to reduce the incidence of certain chronic diseases. The consumption of citrus fruits in the diet, acquires greater interest due to its high content in water and low caloric intake, at the expense of carbohydrates, and with little content in lipids and proteins [2]. Grapefruit is a citrus fruit, which presents high amounts of vitamins, phenolic compounds, and carotenoids [3,4]. However, its seasonality and short life limit its availability and the possibility of enjoying the nutritional and functional properties all year round [5].

Freeze-drying (FD) applied to food preservation is a valuable technique to produces high-quality dehydrated products with very low final water content. The solid state of water during FD protects the primary structure and minimizes the changes to the shape of the product, with a minimal volume reduction [6]. In addition, the reduced temperatures required in the process help to preserve constituents such as minerals, vitamins, and flavonoids, as well as to retain the original color, flavor, and aroma [7,8]. Nevertheless, fruit components as organic acids, such as citric and malic, and low molecular weight sugars, such as sucrose, glucose, and fructose, are responsible for the low value of their glass transition temperature (Tg). This makes the dehydrated product, highly hygroscopic, exhibiting collapse phenomena related to stickiness and caking occurred above the Tg [9]. A common pre-treatment to increase the Tg avoiding these problems is the incorporation of certain high molecular weight solutes as gum arabic or bamboo fiber [10].

The use of this technology in the food industry has reportedly been limited to high added value products, as long processing times and high operation costs are necessary to obtain freeze-dried products with an adequate level of quality. In terms of energy consumption, FD requires almost double the amount of energy to remove 1 kg of water in comparison to conventional dehydration [11]. In this sense, it seems feasible to optimize the process time instead of applying standard conditions that would lead to an unnecessary increase in energy costs. In recent years, several studies dealing with the reduction of FD processing costs have been carried out focusing on the use of combined drying technologies [1,12,13,14,15]. Reducing the initial moisture of the food through partial dehydration with microwaves before freeze-drying seems to be a good alternative means of greatly reducing the drying time [13,15]. The high heating rate of microwaves raises the product temperature quickly and a high vapor pressure is developed inside it, leading to a very rapid transfer of water to the surface of the product. This causes less damage to the product than other heat drying processes, giving a more porous structure inside the dried product and less shrinkage, increased crispiness and lower energy consumption [16]. However, an excessive decrease in humidity before freeze-drying could affect not only the possibility of ice crystal formation during the freezing step, but also the rate of the drying step and, consequently, the high final water content of the obtained product [15]. In this way, the knowledge of drying kinetics is essential for modeling and optimizing a freeze-drying process in order to obtain a shorter process time. 

The aim of this study was to obtain new food ingredients based on freeze-dried grapefruit formulated with high molecular weight solutes (gum arabic and bamboo fiber). To optimize the FD processing, the influence of the water content and the presence of high molecular weight solutes on the grapefruit freeze-drying kinetics were tested; in addition, the nutritional and functional value of the new grapefruit ingredients obtained were evaluated.

## 2. Materials and Methods 

### 2.1. Sample Preparation and Treatments

Grapefruit (*Citrus paradisi* var. Star Ruby) samples were purchased in a local market in Valencia (Spain). Fruits were peeled, washed with distilled water, and resulting pulp was homogenized in a Thermomix (TM 21, Vorwerk, Spain). Three different formulations (F1, F2, and F3) were prepared by adding gum arabic (GA, Scharlau, Spain) and bamboo fiber (BF, VITACEL®, Rosenberg, Germany) to grapefruit pulp in different concentrations (Table 1) previously optimized [17]. The final water content of formulations was reached adding water (F1) or using microwave drying (2 W/g) (F3). Then, the formulated grapefruit purees were freeze-dried. A puree layer (0.5 cm thickness) was placed in a standardized aluminum plate (15 cm diameter and 5 cm height). Consecutively, samples were stored at -45 °C (Liebherr Mediline, LCT2325, Liebherr, Baden-Wurtemberg, Germany) for 24 h before being dried in a Lioalfa-6 Lyophyliser (Telstar, Spain) at 2600 Pa and −56.6 °C for different times, as described in the next section.

### 2.2. Freeze-Drying Kinetics: Mathematical Modeling

In order to evaluate the freeze-drying kinetics of the different formulated purees, the water content of the freeze-dried samples was analyzed after 6, 12, 16, 20, 24, and 30 h. The empirical models of drying were derived from a direct relationship between the average moisture content and drying time. In this work, the freeze-drying curves obtained from the experimental water content of the samples after different process times were fitted to the Page Equation (1), Logarithmic Equation (2) and Midilli-Kucuk Equation (3) models, as proposed by Benlloch-Tinoco et al. [15] for pre-dried and non-pre-dried kiwifruit puree:

(1) $$MR=exp^{-kt},$$

(2) $$MR=a\cdot exp^{-kt}+c,$$

(3) $$MR=a\cdot exp^{-kt}+bt,$$

where,

MR (moisture ratio) = $\frac{M-M_{e}}{M_{0}-M_{e}}$ where,
M: Water mass fraction of the sample at each drying time (g water/g product);
M_e_: Water mass fraction of at the end of the FD process (g water/g product);
M_0_: Water mass fraction of sample before the FD process (g water/g product).
a, b, c and k: Drying constants;
n: Drying exponent;
t: Freeze-drying time (h);

### 2.3. Analytical Determinations: Physicochemical Parameters 

All the analyses described below, except the soluble solid content, were carried out in triplicate on grapefruit freeze-dried powdered samples (G: fresh grapefruit, Grapefruit formulations: F1, F2, and F3). For fresh grapefruit, water content, water activity, and soluble solid content were determined in triplicate.

#### 2.3.1. Water Content

Mass fraction of water (x_w_) was obtained by vacuum drying the samples in a vacuum oven (Vaciotem, J.P. Selecta), at 60 °C during 48 h.

#### 2.3.2. Water Activity

Water activity (a_w_) was measured by using a water activity meter (Aqualab CX-2, Decagon Devices). 

#### 2.3.3. Soluble Solid Content

The soluble solid mass fraction (x_s_) was determined by measuring the °Brix in a previously homogenized sample with a portable digital refractometer Refracto 3PX at 20 °C (Mettler Toledo, Switzerland). 

#### 2.3.4. Porosity

The porosity was obtained from the true and bulk densities, according to Agudelo, et al. [18] and González et al. [19]. In brief, the true density of the samples was calculated from its individual components, in this case, water and carbohydrates, and the bulk density by the ratio mass to volume of the tapped samples.

#### 2.3.5. Color Measurement

Color of the powder samples was measured using a spectrophotometer (MINOLTA, CM3600, Tokyo, Japan) with standard D65 illuminate and 10° visual angle. The powder was placed in a circular aluminium sample holder of 17.7 mm in diameter and 9.53 mm in height. A reflectance glass (CR-A51, Minolta Camera, Japan) was placed between the sample and colorimeter lens. The measurement window was 6 mm in diameter. The results were express in CIEL*a*b* system (CIE, 1986). Chroma, C*_ab_ (saturation) and hue angle, h*_ab_, were also calculated, as defined by: C* = [(a*^2^ + b*^2^)]^1/2^; h* = arctan (b*/a*), respectively. The total color difference (ΔE) taken a non-formulated grapefruit powder as reference (obtained without GA or FB added) was calculated according to: ΔE* = [(ΔL*)^2^ + (Δa*)^2^ + (Δb*)^2^]^1/2^. 

### 2.4. Analytical Determinations: Nutritional Components 

#### 2.4.1. Organic Acids

High performance liquid chromatography-UV detector (HPLC, Jasco equipment, Italy) was used to identify and quantify citric (CA), malic (MA), and tartaric acid (TA), according to Cen et al. [20] and Moraga et al. [8].

#### 2.4.2. Ascorbic Acid and Vitamin C

Ascorbic acid (AA) and vitamin C (ascorbic acid + dehydroascorbic acid) were determined by HPLC-UV detector (Jasco equipment, Italy). The method proposed by Xu et al. (2008) [3] was used to determine the ascorbic acid with some modifications [18].

#### 2.4.3. Vitamin A and E

Vitamin A and E were analyzed using the HPLC-UV method proposed by Munzuroglu, Karatas, and Geckil [21] with some modifications [22].

#### 2.4.4. Flavonoids Determination

The extraction and determination of flavonoids by HPLC-UV detector was carried out according to Moraga et al. [8]. 

#### 2.4.5. Total, Soluble, and Insoluble Dietary Fiber

AOAC enzymatic–gravimetric methods (993.19 and 991.42) were used for soluble dietary fiber (SDF) and insoluble dietary fiber (IDF) analysis [23]. In brief, freeze-dried samples were treated with α-amylase, protease, and amyl glucosidase. The soluble and insoluble fractions were separated by vacuum filtration. Waste from the digests was dried at 100 °C, and ash and protein contents were determined in the residue. Total dietary fiber is the sum of soluble and insoluble dietary fiber fraction [24].

#### 2.4.6. Ash Content and Mineral Composition

Total ash content was determinate following the method 930.05 of the AOAC procedure [23]. A sample of 500 mg was incinerated with high pressure in a microwave oven (Muffle Furnace mls1200, Monroe, USA) for 24 h at 550 °C, and ashes were gravimetrically quantified. The residue of incineration was extracted with HCl (50% *v*/*v*) and HNO3 (50% *v*/*v*) and made up to an appropriate volume with distilled water, where Fe, Cu, Mn, and Zn were directly measured at the suitable wavelength for each element, using standard solutions for calibration purposes. An additional 1/10 (*v*/*v*) dilution was performed in LaCl2 (1.8%, *w*/*v*) for Ca and Mg determination, and CsCl2 (0.2%, *w*/*v*) for Na and K analysis. The methodology followed, previously reported [25] relied on an atomic absorption spectroscopy (AAS) in the Analyst 200 Perkin Elmer equipment (Waltham, USA).

### 2.5. Functional Properties 

#### Antioxidant Capacity

Antioxidant capacity was assessed using the free radical scavenging activity of the samples evaluated with the stable radical DPPH (2,2-diphenyl-1-pierylhydrazyl) [26]. Briefly, 0.1 mL of grapefruit juice sample was added to 3.9 mL of DPPH (0.030 g/L, Sigma–Aldrich, Germany) in methanol. A Thermo Electron Corporation spectrophotometer (Thermo Fisher Scientific, Waltham, Massachusetts, USA) was used to measure the absorbance at 515 nm at 0.25 min intervals until the reaction reached a plateau (time at the steady state). The changes in absorbance were measured at 25 °C. Appropriately, diluted juice samples were used on the day of preparation. The percentage of DPPH was calculated by using Equation (4):

(4) $$\%DPPH=\;\frac{A_{control}-A_{sample}}{A_{control}}\times 100,$$

where *A_control_* is the absorbance of the control (initial time) and *A_sample_* the absorbance of the sample at the steady state. The final results were expressed as millimole trolox equivalents (TE) per 100 g (mmol TE/100 g) using a Trolox calibration curve in the range 6.25–150 mM (Sigma-Aldrich, Darmstadt, Germany).

### 2.6. Statistical Analysis

Non-linear regression analyses were carried out for the estimation of the kinetic parameters. With the aim of evaluating differences in sample behavior, as a function of solute addition, the equations fitted to each individual series and those fitted to different groups of series were statistically compared through the values of statistic E (Equation 5) which was contrasted with tabulated F-Snedecor as a function of the values of DFDR and SFDRi, at 95% significance level [27]:

(5) $$E=\frac{\frac{RSS_{g}-\sum_{i=1}^{n}RSS_{i}}{DFDR}}{\frac{\sum_{i=1}^{n}RSS_{i}}{\sum_{i=1}^{n}FDR_{i}}},$$

where:

RSS_g_: Residual square sum of the function fitted to a group of series;
RSS_i_: Residual square sum of the function fitted to an individual series;
DFDR: Difference between freedom degrees of the residuals of the function fitted to a group of series and the sum of freedom degrees of the residuals of the individual fittings of the series involved in the groups;
FDRg: Freedom degrees of the residuals of the function fitted to a group of series;
FDRi: Freedom degrees of the residuals of the function fitted to an individual series.
SFDRi: Sum of freedom degrees of the residuals of the function fitted to an individual series.

Analyses of variance (ANOVA) were carried out to evaluate the effect of water content and solutes addition. When p value was lower than 0.05, significant differences between samples were assumed. Furthermore, an analysis of the correlation between the antioxidant activity and all the studied compounds, with a 95% significance level, was carried out. ANOVA and Pearson correlations were performed using Statgraphics Centurion XVI.

## 3. Results and Discussion

### 3.1. Freeze-Drying Kinetics of Grapefruit Formulations

Prior formulation, the grapefruit raw material was characterized. The average values (and standard deviation) of water and soluble solids mass fraction (°Brix) and water activity of fresh grapefruit used as raw material were x_w_ = 87.82 (0.02) g_w_/100g, x_s_ = 11.0 (0.2) g_ss_/100 g and a_w_ = 0.989 (0.003). These values are coincident with those reported by other authors [28].

**Figure 1:**
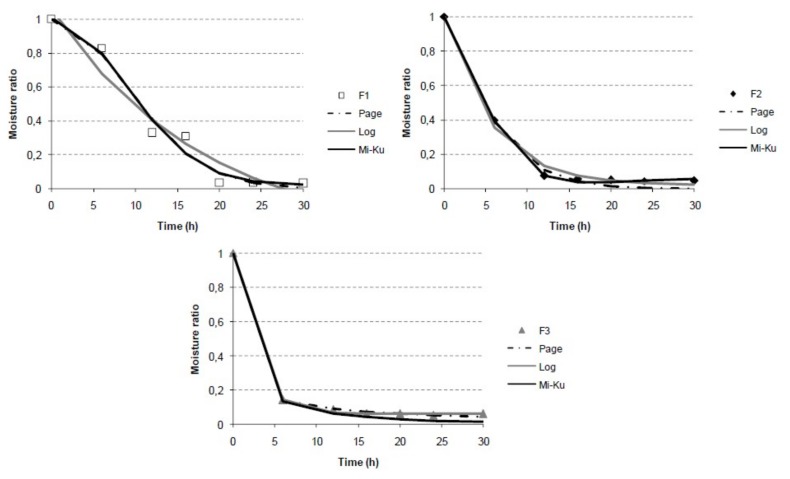
Experimental and modeled freeze-drying curves (Page, logarithmic, and Midilli-Kucuk models) for grapefruit formulations (F1, F2, and F3).

The contribution of the initial water content and solute addition to the freeze-drying kinetics of grapefruit puree was evaluated. To this end, the freeze-drying curves of all the formulated samples were obtained by plotting the moisture ratio vs. time. In turn, the experimental data were fitted to the Page, logarithmic, and Midilli-Kucuk models. Figure 1 shows the experimental and modeled M_R_ behavior of all the studied grapefruit formulated samples (F1, F2, and F3) during the freeze-drying process. The kinetic parameters and the accuracy of the fit determined for the three models and all the samples are presented in Table 2. These models coincided well with the experimental data, as can be seen from the obtained adjusted regression coefficient (R^2^) and the root mean square error (RMSE) values. The best fit (higher R^2^) was Midilli-Kucuk for F2 and F3, and Page for F1. The values obtained for all the kinetic parameters were in the range commonly observed for different agricultural products [14,29]. In these models, *k* is the drying rate constant [30] and the *n* parameter has an effect of moderating the time and gives better results for the prediction of moisture loss from composite materials [30]. An increase in *k* may compensate for a decrease in *n*, leading to the drying kinetic behaving in a similar way. Both parameters, *k* and *n*, have been correlated to different process variables, such as initial moisture content, among others [31].

In this study, the drying constant *k* varied from 0.0070–1.2 h^−1^ for the Page model, 0.0533–0.4015 h^−1^ for the logarithmic model, and 0.0069–0.8671 h^−1^ for the Midilli-Kucuk model, and the parameter *n* varied from 0.28–1.95 for the Page model and 0.47–1.97 for the Midilli-Kucuk model (Table 2). The drying constant decreases and *n* increases when the initial moisture content rises and the solute content drops. 

The possible existence of statistical differences in the drying kinetics of all samples considered in the present study was evaluated by considering the Page and Midilli-Kucuk models. To this end, the models fitted to each individual series and the models fitted to different groups of data series were compared through the value of statistic E and the tabulated F-Snedecor as a function of the values of DFDR and SFDRi Equation (5), at 95% significance level. Table 3 shows the groups of experimental series in which the statistical differences between the fitted Page and Midilli-Kucuk functions were analyzed. Significant differences were obtained in the comparison of the three formulations studied series (E>F-Snedecor). It can be concluded that the initial water and solute content had a significant impact (E>F-Snedecor) on the FD kinetic of samples when they were described both, by the Page and Midilli-Kucuk models. 

**Figure 2:**
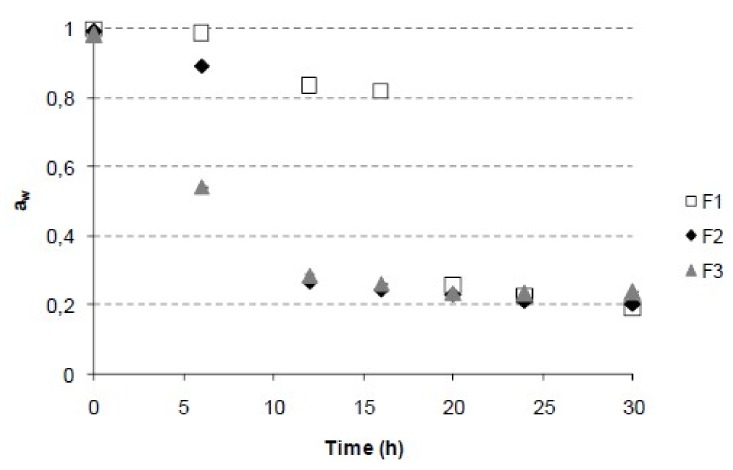
Water activity evolution along kinetic freeze-drying of grapefruit formulations.

Figure 2 shows the water activity evolution during the freeze-drying process. The highest a_w_ losses were during the 12 first hours in F2 and F3 and during the first 20–24 hours in F1. Thus, the a_w_ values at the end of the kinetic study (30 h) were different for each formulation. F3 (minor initial water content) exhibited higher a_w_ (0.235) comparing with the rest that they were in the range 0.186–0.202. This may be related to the cryoprotective effect of the partial dehydration of the samples, resulting in a smaller amount of ice formed in the sample during the freezing step [15].

The Page, logarithmic, and Midilli-Kucuk models are semi-theoretical, which implies that they derive a direct relationship between the water content and the drying time [30]. In order to predict the time required for the freeze-drying in each case, the Page model was used for each sample. The end point was estimated as the average of the moisture reached in the final stretch of the kinetics in which no variation of humidity or a_w_ was observed. According to this, adequate freeze-drying times for F1, F2, and F3 were 24, 16, and 18 h, respectively. 

### 3.2. Physicochemical Properties of Freeze-Dried Formulations

Table 4 shows water content, water activity, porosity, and color coordinates of each final grapefruit formulated FD powder. There were no significant (*p* < 0.05) differences among samples according to water content. However, F2 and F3 formulations, showed significantly (*p* < 0.05) higher values of water activity. 

In general, porosity values are similar to other grapefruit freeze-dried [18]. A greater porosity corresponds to a more free-flowing powder. The porosity increased slightly when gum Arabic and bamboo fiber was added just as when water was incorporated in previous freeze-drying. However, there were no significant (*p* > 0.05) differences among final samples.

With respect to color parameters, samples with higher solutes added in the formulation (F2 and F3) presented significantly (*p* < 0.05) higher L*values with respect to G and F1, being samples lighter. Agudelo et al., 2016, also observed this behavior [18]. The use of the microwave to reduce the water content previously to freeze-drying decreased significantly the values of a*, b*, h*, and C*, above all in F3 because the microwave pre-treatment was the longest and it provoked a brownish tone. Similar color differences with respect to grapefruit powder were found between F1 and F2 and close to 3. ΔE >3 implies perceptible color difference by the human eye [32]. Increase in E for F1 and F2 was less than a unit thus practically imperceptible for consumers. Nevertheless, F3 showed color differences with respect to grapefruit powder higher than 3, which reflected different tonalities to characteristic grapefruit color perceptible by consumers, possible due to a large microwave treatment applied.

### 3.3. Composition of Freeze-Dried Formulations

In Table 5 it can be observed the mean values (with standard deviation) of total fiber, organic acids (tartaric, malic, and citric acid), and vitamins A, C, and E of studied powder grapefruit formulated samples. Citrus fruit stands out for its high content of soluble fiber [2,5]. It is widely recognized that insufficient consumption of fiber in Western societies is closely related to certain health problems, thus the intake of fiber is highly recommended. Fiber intake can be obtained from foods that contain it naturally (such as fruits and vegetables) or eating those foods to which fiber has been added as a functional ingredient [24]. The incorporation of bamboo fiber in grapefruit formulations increase significantly (*p* < 0.05) total fiber content, as can be observed in grapefruit powder samples F2 and F3. Thus the use of these powders as a potential bioactive food ingredient may be proposed as an interesting option. 

Powder grapefruit formulated samples were characterized by high content of citric acid (CA) as the major organic acid followed by malic (MA) and then tartaric acid (TA). This sequence was also observed in grapefruit [4,22] and in other citric fruit [5] by other authors. Comparing the novel powder grapefruit formulated samples, F1 presented the lowest concentration of CA, and F2 the lowest of TA and MA. F3 was the most similar to fresh grapefruit. 

As can be observed in Table 5, among studied vitamins, the vitamin most abundant in grapefruit powder was vitamin C, as in other products obtained from this fruit, for example, juice [4], jams [23], or powders without solutes [8]. Vitamin C is used as reference in different industrial processes since its presence ensures a high nutritional quality of the final product due to its easy degradation [33]. In order to compare this possible degradation in the process of obtaining the samples considered in this study, all of them with different water and solutes content, the vitamin C values were expressed as mg/100 g of grapefruit solutes such as in another author works [18,34]. The values for fresh grapefruit (G), and formulated samples, F1, F2, and F3, were 380 (23), 334 (8), 381 (13), and 377 (4) mg/100g grapefruit solutes, respectively. Therefore, F2 and F3 were the most similar to G according to vitamin C, without observed losses of this vitamin in spite of microwave treatment. However, the losses of vitamins A and E of the proposed formulations of grapefruit powder were significant (*p* < 0.05).

The 98% of the total flavonoids present in grapefruits are flavanones [35,36,37]. Table 6 shows the flavonoids of grapefruit powder and studied formulations taking into account the major flavanones (naringin, NAR; narirutin, NAT; hesperidin, HES; neohesperidin, NEOH; didymin, DID; and poncirin, PON), flavones (naringenin, NAG), and flavonols (quercetin, QUER)). The total flavonoid content analyzed in G was in good agreement with previous findings of the total phenol content (174 mg of gallic acid/100 g fresh grapefruit for powder rehydrated, Moraga et al. [8]). NAR was the most abundant flavonoid in grapefruit powder, followed by NAT, and NAG, results that coincided closely with other studies [35,36,37,38]. The values found for every compound were in the same range as those reported in other publications for grapefruit [3]. In general, the proposed formulations showed significantly (*p* < 0.05) lower total content of flavonoids than the rest. However, F1 and F3 presented higher values of NAG and DID, respectively. Maltodextrins and gums are added during the production of food powders in order to act as encapsulating or wall materials, contributing to keep the desired functional properties in the finished product, such as stability against oxidation, ease of handling, improved solubility, controlled release, and extended shelf-life [39]. As aforementioned for vitamin C, this protector effect can be observed when the values are expressed as mg/100g of grapefruit solutes. In the case of total flavonoids, for G, F1, F2, and F3 were 1992 (74), 1849 (55), 2089 (99), and 2155 (52) mg/100g grapefruit solutes, respectively. In this way, F3 showed the highest flavonoid content so gum arabic and bamboo fiber present in this formulation keep these compounds in grapefruit powder, in spite of large microwave treatment to reach the water content established.

**Figure 3:**
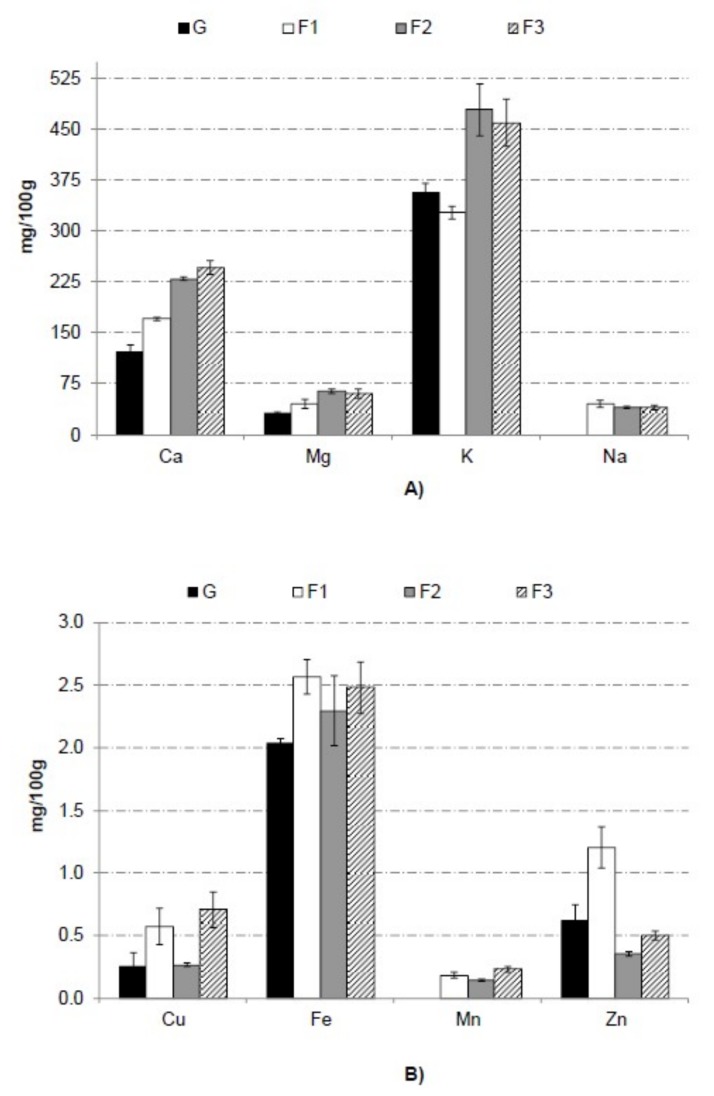
Macrominerals (**A**) and microminerals (**B**) content of grapefruit powder (G) and each formulation (F1, F2, and F3). Letters indicate homogeneous groups established by the ANOVA (*p* < 0.05) for each mineral.

Figure 3 shows macrominerals (A) and microminerals (B) content of grapefruit powder and each formulation. Grapefruit mineral content was similar to values of other authors [40] for grapefruit. Potassium is the main mineral in grapefruit. This element is involved in the regulation of the water and electrolyte balance and the acid-base balance in the body [41]. In addition to potassium, sodium is also responsible for regulation of the water and electrolyte balance. There is much less sodium than potassium in grapefruit, which is important information for those who have problems with blood pressure regulation and conditions associated with hypertension [42]. 

In general, the use of gum arabic and bamboo fiber in formulation increase significantly (*p* < 0.05) mineral content in grapefruit powder obtained by freeze-drying. By the intake of 100 g of F2 or F3 sample, the increase of K is approximately 30% compared with G. Citrus fruits, in comparison with other fruits, such as apples, pears, melons, peaches, plums, mangoes, and bananas, are a valuable source of calcium, which plays an important role in building hard, strong bones [42]. Furthermore, the addition of gum arabic and bamboo fiber increased the calcium content in 40%, 88%, and 101% for F1, F2, and F3, respectively. Iron increase significantly (*p* < 0.05) in formulations with gum arabic and bamboo fiber around 23%. One of the most important minerals in human nutrition is iron according to Latham (2002) [43]. Iron is an essential element for almost all living organisms as it participates in a wide variety of metabolic processes, including oxygen transport, deoxyribonucleic acid synthesis, and electron transport [44]. The increase in Fe for studied solutes addition is very valuable information, especially for people with high iron requirements, i.e., women of childbearing age and pregnant women. Magnesium is 92% higher in samples F2 and F3 with respect to G so these formulations presented a high magnesium content, which is particularly important in the diet of older people suffering from depression, insomnia, or recurrent muscle cramps, as well as in physically active individuals [45]. Manganese was not detected in G, however F1, F2, and F3 showed 0.182, 0.139, and 0.230 mg/100 g as a consequence of incorporation of gum arabic and bamboo fiber in formulation.

### 3.4. Antioxidant Capacity of Freeze-Dried Formulations

Mean values (and deviation) were 4.254 (0.014), 4.181 (0.004), 4.16 (0.02), and 3.96 (0.18) mg TE/100 g for G, F1, F2, and F3, respectively. F3 presented significant differences with respect to the others. However, when the values were expressed as mg/100 g of grapefruit solutes they were 4.422 (0.015), 4.317 (0.004), 5.38 (0.02), and 4.8 (0.2) for G, F1, F2, and F3, respectively. Thus, the addition of solutes maintained largely the antioxidant capacity even when microwave treatment is applied (sample F3).

In order to explain the influence of the different compounds quantified in this study on the antioxidant capacity of the samples, correlation statistical analyses were performed. Total flavonoids played a major role in the antioxidant capacity of grapefruit powders (0.8449, *p* < 0.05), followed by the vitamin A (0.7301, *p* < 0.05) and the vitamin C (0.7129, *p* < 0.05). Other studies [4,46] confirm the existence of a positive relationship between the phenolic content of a fruit and its antioxidant capacity. Fruits with high antioxidant activity generally contain a great quantity of antioxidant substances, especially phenolic compounds, and specifically flavonoids [47].

## 4. Conclusions

The best FD kinetic model fit on grapefruit powders was Midilli-Kucuk for F2 and F3, and Page for F1. Adequate freeze-drying times for F1, F2, and F3 were 24, 16, and 18 h, respectively. The initial water and solute content had a significant impact on the FD kinetic of samples. The application of a mild microwave drying pre-treatment, as it is 2 W/g to reach no less than 71 g water/100 g mixture (F3), leads to a faster dehydration of grapefruit puree samples during freeze-drying processing. Although this formulation (F3) presented changes in characteristic grapefruit color of powders, it showed higher antioxidant capacity as a consequence of gum arabic and bamboo fiber protector effect on flavonoids and vitamin C, in comparison with the other formulations (F1 and F2). The new food ingredients, obtained and characterized, could be of great interest for the food industry to be offered as bioactive compounds in order to develop foods enriched in natural fibers and/or micronutrients as well as to improve food antioxidant capacity. Although all these powdered products apparently showed good physical properties, subsequent studies are planned to be carried out as for a sensory evaluation and to know their solubility, hygroscopicity, or flowability.

## Figures and Tables

**Table 1:** Composition of each formulation (F1, F2, and F3) of grapefruit.

Formulation	Fruit Puree (g)	Gum Arabic (g/100 g_fresh fruit_)	Bamboo Fiber (g/100 g_fresh fruit_)	x_w_ g_w_/100 g_F_
F1	100	0.73	0	94
F2	100	2.6	0.32	80
F3	100	3.8	0.47	71

**Table 2:** Values of the freeze-drying kinetic parameters obtained for the three grapefruit formulations (F1, F2, and F3) when the Page, logarithmic and Midilli-Kucuk models were used to fit the experimental data. Adjusted regression coefficient (Adj. R^2^) and root mean square error (RMSE) values.

Sample Code	Model			
		*Page*	*Logarithmic*	*Midilli-Kucuk*
***F1***	**Model constants**	k: 0.0070 n: 1.9515	a: 1.3825 k: 0.0533 c: −0.3251	a: 1.0092 k: 0.0069 n: 1.9757 b: 0.0006
	**Adj. R^2^**	97.33	91.61	95.67
	**RMSE**	0.0427	0.0774	0.0040
***F2***	**Model constants**	k: 0.1027 n: 1.2363	a: 0.9906 k: 0.1788 c: 0.1672	a: 1.0000 k: 0.0550 n: 1.5989 b: 0.0019
	**Adj. R^2^**	99.08	98.74	99.89
	**RMSE**	0.0245	0.0244	0.0062
***F3***	**Model constants**	k: 1.2000 n: 0.2813	a: 0.9387 k: 0.4015 c: 0.0612	a: 1.0000 k: 0.8671 n: 0.4670 b: 0.0014
	**Adj. R^2^**	99.92	99.93	99.98
	**RMSE**	0.0059	0.0044	0.0025

**Table 3:** Statistical comparison among grapefruit formulations (F1, F2, and F3) obtained according to Table 1 in terms of the fitted models.

	Page Model	Midilli-Kucuk Model
F1	x	x
F2	x	x
F3	x	x
SCRG	0.33	0.33
SCRi	0.0276	0.0211
GLRG	19	17
DGLR	4	8
SGLR	15	9
E	41.612 *	16.506 *
F (95%)	3.056	3.230

SCRG: sum of residual squares of the general function; SCRi: sum of residual squares of function i;.GLRG: degrees of freedom of the residual of the general function; DFDR: Difference between freedom degrees of the residuals of the function fitted to a group of series and the sum of freedom degrees of the residuals of the individual fittings of the series involved in the groups; SFDR: Sum of freedom degrees of the residuals of the function fitted to an individual series; E: statistic E; F: tabulated F-Snedecor. * significant differences at 95% of significant level

**Table 4:** Mean values (and standard deviation) of color coordinates (L*, a*, b*), hue angle (h*), chrome (C*), color differences (ΔE*), porosity, water content, and water activity of grapefruit powder (G) and each grapefruit formulations (F1, F2, and F3).

Parameter	G	F1	F2	F3
Water content (g/100 g)	4.4 (0.2) ^a^	4.7 (0.2) ^a^	4.9 (0.5) ^a^	4.9 (0.3) ^a^
Water activity	0.266 (0.003) ^c^	0.286 (0.003) ^b^	0.293 (0.003) ^a^	0.294 (0.003) ^a^
Porosity	0.672 (0.006) ^b^	0.6975 (0.0019) ^a^	0.684 (0.004) ^ab^	0.690 (0.004) ^a^
L*	70.89 (0.1700) ^c^	70.7 (0.4) ^c^	72.9 (0.5) ^b^	75.48 (0.12) ^a^
a*	15.397 (0.018) ^a^	14.2 (0.2) ^c^	14.54 (0.072) ^b^	12.2 (0.08) ^d^
b*	33.12 (0.0200) ^a^	30.3 (0.4) ^b^	30.170 (0.104) ^b^	24.72 (0.16) ^c^
h*	65.07 (0.0200) ^a^	64.95 (0.07) ^a^	64.27 (0.08) ^b^	63.9 (0.2) ^c^
C*	36.53 (0.0300) ^a^	33.5 (0.5) ^b^	33.49 (0.12) ^b^	27.53 (0.13) ^c^
E*	-	3.1 (0.5) ^c^	3.72 (0.15) ^b^	10.12 (0.15) ^a^

The same letter in superscript within rows indicates homogeneous groups established by ANOVA (*p* < 0.05).

**Table 5:** Mean values (with standard deviation) of total fiber (g/100 g) organic acids (mg/100 g), and vitamins (mg/100 g) analyzed in grapefruit powder (G) and each grapefruit freeze dried formulation (F1, F2, and F3).

Compound	G	F1	F2	F3
Total fiber	25.5 (0.7) ^b^	19.8 (0.2) ^c^	36.5 (0.8) ^a^	36.7 (0.3) ^a^
Tartaric acid	1645 (54) ^a^	1419 (156) ^ab^	1304 (23) ^b^	1535 (23) ^ab^
Malic acid	2310 (16) ^a^	1727 (31) ^b^	1623 (39) ^c^	1788 (39) ^b^
Citric acid	2943 (8) ^a^	1964 (70) ^b^	2228 (163) ^b^	2805 (78) ^a^
Vitamin C	356 (8) ^a^	323 (8) ^b^	313 (11) ^b^	291 (3) ^c^
Vitamin A	7.7 (0.3) ^a^	4.8 (0.4) ^c^	6.0 (0.3) ^b^	3.8 (0.5) ^c^
Vitamin E	1.7 (0.2) ^a^	0.58 (0.05) ^c^	1.20 (0.08) ^b^	0.38 (0.07) ^c^

The same letter in superscript within rows indicates homogeneous groups established by ANOVA (*p* < 0.05).

**Table 6:** Mean values in mg/100 g (with standard deviation) of flavonoids (narirutin, naringin, hesperidin, neohesperidin, didymin, poncirin, naringenin, and quercetin) analyzed in grapefruit powder (G) and each grapefruit freeze dried formulation (F1, F2, and F3).

Flavonoid	G	F1	F2	F3
NAT	285 (9) ^a^	259 (6) ^b^	255 (5) ^b^	249 (6) ^b^
NAR	1516 (58) ^a^	1414 (42) ^b^	1410 (41) ^b^	1260 (59) ^c^
HES	26 (3) ^a^	22.4 (1.0) ^b^	21 (3) ^bc^	18.6 (1.1) ^c^
NEOH	5.6 (0.4) ^a^	4.0 (0.5) ^b^	5.6 (0.5) ^a^	5.4 (0.4) ^a^
DID	13.9 (1.4) ^b^	14.3 (1.2) ^ab^	13.6 (0.3) ^b^	16.0 (0.6) ^a^
PON	16.9 (0.5) ^a^	15.5 (0.7) ^b^	13.6 (0.7) ^c^	12.8 (0.4) ^c^
NAG	51.5 (1.3) ^b^	60 (3) ^a^	51 (5) ^b^	54 (4) ^ab^
QUER	1.09 (0.15) ^a^	1.20 (0.08) ^a^	0.341 (0.014) ^c^	0.67 (0.07) ^b^
Total Flavonoids	1916 (62) ^a^	1791 (46) ^b^	1771 (37) ^b^	1618 (67) ^c^

The same letter in superscript within rows indicates homogeneous groups established by ANOVA (*p* < 0.05). NAT: narirutine, NAR: naringin, HES: hesperidin, NEOH: neohesperidin, DID: didymin, PON: poncirin, NAG: naringenin, and QUER: quercetin.
